# Retraction Note: Childhood iron deficiency anemia leads to recurrent respiratory tract infections and gastroenteritis

**DOI:** 10.1038/s41598-021-90018-8

**Published:** 2021-05-11

**Authors:** Jayaweera Arachchige Asela Sampath Jayaweera, Mohammed Reyes, Anpalaham Joseph

**Affiliations:** 1 Department of Microbiology, Faculty of Medicine and Allied Sciences, Rajarata University of Sri Lanka, Saliyapura, Sri Lanka; 2 Department of Pediatrics, Faculty of Medicine and Allied Sciences, Rajarata University of Sri Lanka, Saliyapura, Sri Lanka

Retraction of: *Scientific Reports* https://doi.org/10.1038/s41598-019-49122-z, published online 02 September 2019

The Editors have retracted this Article.

Following publication, concerns were raised regarding the ethics approval for this study. Contrary to the statement in the Article, the Authors were unable to provide appropriate documentation confirming that ethics approval was obtained for this study.

All Authors agree with the retraction and its wording.

